# Sexual Risk Behaviors of Adolescents with Type 1 Diabetes in Comparison with Their Peers

**DOI:** 10.3390/children9010020

**Published:** 2021-12-29

**Authors:** Betina Kandyla, Artemis Tsitsika, Alexandra Soldatou, Chara Tzavara, Spyridon Karanasios, Kyriaki Karavanaki

**Affiliations:** 1 Diabetes and Metabolism Clinic, Second University Department of Pediatrics, “P. & A. Kyriakou” Children’s Hospital, National and Kapodistrian University of Athens School of Medicine, 115 27 Athens, Greece; alsoldat@med.uoa.gr (A.S.); spyroskara97@yahoo.gr (S.K.); kkarav@yahoo.gr (K.K.); 2 Adolescent Health Unit (A.H.U.), Second University Department of Pediatrics, “P. & A. Kyriakou” Children’s Hospital, National and Kapodistrian University of Athens School of Medicine, 115 27 Athens, Greece; info@youth-health.gr (A.T.); htzavara@med.uoa.gr (C.T.)

**Keywords:** sexuality, chronic disease, diabetes mellitus, hazardous behaviors, teenagers

## Abstract

Background: Adolescents with type 1 diabetes mellitus (T1D) may differ from peers regarding sexual risk behaviors. Objectives: To explore sexual risk behaviors of adolescents with T1D in comparison with peers. Materials and methods: The subjects were 174 adolescents, 58 adolescents with T1D (mean ± SD age 16.3 ± 2.0 yrs, disease duration 6.7 ± 3.5 yrs and HbA1c:8.0 ± 1.3%) and 116 without (matched 1:2). Anonymous, self-reported questionnaires were used to evaluate sexual education and behaviors. Results: Fewer adolescents with T1D than those without had a sexual experience (74.1% vs. 87.4%, *p* = 0.033), with similar age of sexual debut. Among adolescents with T1D, ≥2 risky behaviors were observed less frequently than adolescents without T1D (8.62% vs. 23.27%, *p* = NS respectively) and in fewer girls than boys in both adolescents with T1D (0% vs. 18.5%, *p* = NS) and adolescents without T1D (11% vs. 44%, *p* = 0.022). Adolescents with T1D with ≥2 risky behaviors were older (*p* = 0.031), younger at first sexual intercourse (*p* = 0.031), with higher maternal education (*p* = 0.039). Early sexual debut was associated with higher maternal education (*p* = 0.014) and HbA1c (*p* = 0.049). Most adolescents without T1D with ≥2 risky behaviors were boys and older than peers. Conclusions: Adolescents with T1D and females were more cautious than adolescents without T1D regarding sex. The associations of increased risky behaviors with male gender, older age, younger age at sexual debut and higher maternal education in adolescents with T1D merit further investigation.

## 1. Introduction

Type 1 diabetes is a demanding chronic disease that requires a complex regimen of insulin, healthy diet and exercise. It affects everyday life of people with T1D and has a particular impact on adolescents and their caregivers [1]. Glycemic control usually deteriorates during adolescence. Since diabetes control is not only an individual responsibility, inevitable parental involvement in diabetes management and the adolescents’ lives may generate further parental–child conflicts [2,3]. As adolescents with diabetes frequently face psychosocial issues and have an increased need for peer acceptance, they are considered at high risk of risky behaviors and substance abuse [4].

A child’s transition to adulthood during adolescence is achieved through the development of a healthy body image and self-identity, establishment of emotional and economic independence from his/her parents and management of his/her sexuality [5]. This transition becomes more complicated in adolescents with any chronic disease, increasing the risk of hazardous behaviors [6]. Risky sexual behaviors among adolescents include (a) the presence of multiple (≥4) sexual partners, (b) older age of sexual partner (≥6 years), (c) intoxication with alcohol before sex, (d) having sex with no consent and (e) unprotected sex [7].

Although individual, family and societal protective and predisposing factors have been previously described [8,9], no single model can fully explain adolescent risk-taking behaviors [5]. Given identified predisposing factors for chronically ill adolescents such as lower self-esteem, decreased peer acceptance, and increased life stress, they are considered at increased risk for hazardous behaviors [5]. On the other hand, risky behaviors may affect diabetic control and lead to the development of acute and chronic complications [10]. Alcohol use can have a significant negative impact on blood glucose levels by increasing glycemic variation, increasing the risk for hypoglycemia, and compromising the ability to detect hypoglycemia [11,12,13]. Cigarette smoking increases HbA1c levels [14,15] and the risk for diabetic nephropathy and retinopathy [16,17]. Illicit drugs increase the risk for hypo- or hyperglycemia and ketoacidosis [18]; in addition, impaired judgment compromises the ability to perform self-management tasks. Unprotected sex increases the risk for unplanned and/or early pregnancy, obstetrical complications, ketoacidosis and sexually transmitted diseases [13,19].

Although health care providers should offer routine counseling for adolescents with T1D about personal safety and glycemic control, there are limited previous studies on the factors associated with the development of risky behaviors in adolescents with T1D [20,21] and most of them did not include control groups.

The aims of this study were to assess the presence of sex-related risky behaviors among Greek adolescents with T1D in comparison with their peers and investigate potential predisposing factors, such as age, gender, diabetes duration, glycemic control and parental educational status.

## 2. Materials and Methods

### 2.1. Materials

Adolescents and young adults with T1D aged 14–21 years regularly followed at the Diabetes and Metabolism Clinic of the Second Department of Pediatrics, National and Kapodistrian University of Athens, “P. & A. Kyriakou” Children’s Hospital were recruited prospectively from 2016 to 2019. The study also included 116 adolescent students (controls) matched for age, gender and socioeconomic level, who were recruited from grades 9–12 school level on a 1:2 ratio, with the single exclusion criterion of suffering from any acute or chronic disease.

In Greece, children 6–12 years old attend primary school (grade 1–6), adolescents 13–15 years old attend junior high (intermediate) school (grade 7–9) and adolescents 16–18 years old attend senior high (secondary) school (grade 10–12). Students attending Grades 9–12 were invited to participate in the study.

The study was approved by the Ethics Review Board of both the “P. & A. Kyriakou” Children’s Hospital in Athens, Greece, and the Hellenic Ministry of Education and Religious Affairs. Informed consent for study participation was obtained from all parents/legal guardians of eligible participants prior to participation in the study.

### 2.2. Methods

Demographic data of all participants were collected, including place of residence, parental educational level and socioeconomic status. All participants without T1D self-reported their somatometric parameters (weight, height, BMI).

For adolescents with T1D the somatometric parameters (weight, height, BMI), blood pressure and heart rate were measured and recorded by a nurse or a medical doctor on the day of participation in the study during their appointment at the Diabetes Unit.

All adolescents with T1D completed a detailed personal medical questionnaire, including T1D duration, age at diabetes diagnosis, type of insulin therapy. The mean HbA1c over the past year was recorded from their medical records as an index of glycemic control.

Furthermore, in order to minimize any potential reporting bias, all study participants self-completed two anonymous questionnaires to evaluate: (1) parental education, occupation and marital status, (2) sexual activity and behavior, (3) contraceptive use, (4) psychosocial factors and (5) sources accessed for sexual information.

Controls were initially informed about the objectives of the study by a trained psychologist or medical doctor and then responded to the questionnaire following the instructions provided. Self-completed questionnaires were administered on separate slips of paper and filled out by participating adolescents in class.

T1D adolescents were informed about the objectives of the study by a trained medical doctor and filled out the questionnaires in a private room on the day of attendance at the Diabetes Unit.

The first 72-item questionnaire has been used previously in studies of sexual activity and contraception methods among healthy Greek adolescents [22,23] and assessed the following:

(a) Socio-demographic variables. Gender and exact age were reported along with the parents’ educational attainment, measured by the highest qualification earned by either parent.
(b) Sexual activity. For the purposes of the study, sexual experience was defined as any sexual contact (varying from holding hands, hugging and mouth kissing to oral/anal sex), excluding vaginal sexual intercourse, on at least one occasion. Sexual intercourse was defined as vaginal sexual intercourse on at least one occasion.
(c) Contraceptive use. Study participants were requested to report the contraceptive methods used during sexual contact and/or intercourse. The contraceptive methods assessed included the following: (1) no protection applied during sexual contact, (2) withdrawal of penis prior to ejaculation, (3) avoiding sexual intercourse during possible ovulation days (“calendar method”), (4) male condom use, (5) oral contraceptive use and (6) morning after pill (“emergency contraception”). Before filling in the questionnaire, the study participants attended a presentation about sexual risky behaviors, sexually transmitted diseases and contraceptive methods, explaining that some of them are not effective or not recommended, such as no protection, withdrawal of penis prior to ejaculation, calendar method and emergency contraception.
(d) Other psychosocial factors evaluated were: (1) family status, (2) traumatic or other major life events, (3) peer influence upon sexual initiation and (4) being forced to have a coital experience.

The second questionnaire used was the Youth Self-Report [24] (11–18 years) (YSR/11–18) to access psychosocial status (emotional problems and behaviors) of Greek adolescents. The Youth Self Report (YSR) is a widely used instrument for the assessment of adolescent competencies and behavioral problems. The reliability and validity of the YSR were originally documented by Achenbach (1991a), and the YSR has been translated and validated in a Greek adolescent population [25].

The YSR consists of two parts; the first assesses adaptive behavior and forms two scales, activities and social, as well as a total competence scale. The second consists of 112 items that contribute to the scale scores and 16 items that refer to socially desirable skills, which do not contribute to the scale scores. Self-rating is based on a Likert type scale from 0 to 2 (0 = not true, 1= somewhat or sometimes true, 2 = very true or often true) for the preceding 6 months. A summary score is obtained for eight syndrome scales: withdrawal, somatic complaints, anxiety/depression (which together form the broadband internalizing problems scale), delinquent behavior and aggressive behavior (which together form the broadband externalizing problems scale), social problems, thought problems and attention problems. Total problem is the sum of scores on all items.

### 2.3. Statistical Analysis

The Kolmogorov–Smirnov test was used for the estimation of normal distribution of quantitative variables. Quantitative variables are expressed as mean values (SD) or as median values (interquartile range). Qualitative variables are expressed as absolute and relative frequencies. For the comparison of proportions, chi-square tests and Fisher’s exact tests were used when necessary. Student’s *t*-tests were computed for the comparison of mean values when the distribution was normal and the Mann–Whitney test for the comparison of median values was used when the distribution was not normal. Linear regression analysis in a stepwise method (*p* for entry 0.05, *p* for removal 0.10) was used in order to find independent factors associated with age at first sexual experience. All *p* values reported are two-tailed. Statistical significance was set at 0.05 and analyses were conducted using the SPSS statistical software (version 19.0).

## 3. Results

The study group included 174 adolescents aged between 14 and 21 (mean age 15.9 ± 1.6) years. Of those, 58 T1D adolescents (29 boys and 29 girls, mean age 16.3 ± 2 years, disease duration 6.7 ± 3.5 years and mean 12 month HbA1c 8.0 ± 1.3%) were compared to 116 matched adolescents without T1D (58 boys and 58 girls, mean age 15.8 ± 1.4 years).

Compared to their peers, a statistically significantly lower percentage of adolescents with T1D had a sexual experience (74.1% vs. 87.4%, *p* = 0.033) (Table 1). A lower percentage of girls than boys had sexual experience in both study groups. Specifically, 89.6% (52/58) of boys without T1D and 79.3% (23/29) of boys with T1D reported having a sexual experience. Among girls, 77.5% (45/58) of those without T1D and 58.6% (17/29) of those with T1D had a sexual experience (*p* = 0.065), which was marginally statistically non-significant. The mean (±SD) age of a first sexual experience was similar for both groups; 14.7 (±1.5) years for the adolescents without T1D and 14.5 (±2.3) years for those with T1D.

When estimating the difference between the age at first sexual experience and the age at first sexual intercourse, no statistically significant difference between adolescents with and without T1D was observed (T1D adolescents vs. controls: 0.60 ± 1.35 vs. 0.51 ± 1.09 years, *p* = 0.799). Moreover, no significant difference between genders was observed (0.60 ± 1.31 vs. 0.40 ± 0.73 years, *p* = 0.582, for the total group of boys and girls, respectively).

Risky behaviors in sexually active adolescents for both groups are shown in Table 2. One third of the participants in both groups reported having their first sexual intercourse with an older partner. No significant difference in the proportion of adolescents that had their first sexual intercourse with an older partner was observed between the adolescents without T1D (29.3%) and those with TID (31.0%, *p* = 0.265). In most cases, (adolescents without T1D: 79.1% vs. adolescents with T1D 94.8%, *p* = 0.519) the age difference between sexual partners was under 5 years.

Most adolescents in both groups reported having had ≤2 sexual partners; specifically, 57.5% of the adolescents without T1D and 68.2% of those with T1D (Table 2). Although not statistically significant, proportionally fewer adolescents with T1D reported forced sex than those in the comparison group (4.3% vs. 15.7%). Furthermore, girls in both groups reported fewer sexual partners than boys. Although reported alcohol consumption before sexual intercourse was similar for both groups, fewer adolescents with T1D than adolescents without T1D reported alcohol intoxication prior to sexual intercourse, and all cases were boys (4.3% vs. 20%, *p* = 0.0156).

Finally, despite not being statistically significant, compared to adolescents with T1D, a three times greater proportion of adolescents in the comparison group reported unprotected sex (6.66% vs. 18.9%, *p* = 0.339).

In Table 3, it is shown that the prevalence of risky behaviors was markedly lower among girls of both groups, and especially among the T1D group, no girl and very few boys had risky behaviors.

The prevalence of ≥2 risky behaviors was three times higher in the adolescents without T1D than in the T1D group (23.2% vs. 8.2%, respectively).

In Table 4, the characteristics of T1D adolescents with ≥2 risky behaviors are shown. T1D adolescents with ≥2 out of 5 risky behaviors were all boys (*p* = 0.045), of older age (*p* < 0.031) and younger at their first sexual intercourse (*p* = 0.031), compared to T1D adolescents with <1 risky behavior. Risky behavior in the T1D group was associated with higher maternal education (university/postgraduate studies) (*p* = 0.039). T1D adolescents with risky behaviors did not differ from those without in terms of HbA1c levels, age at diagnosis and disease duration.

Multiple linear regression analysis of potential factors associated with age at first sexual experience among T1D adolescents revealed associations with higher maternal education (b = −1.47, *p* = 0.014) and poor glycemic control [HbA1c (b = −0.63, *p* = 0.049)].

In the comparison group, adolescents with ≥2 risky behaviors were older in age (*p* < 0.006) and predominantly boys (*p* = 0.001), compared to those without (Table 5). Risky behaviors in the adolescents without T1D group were not associated with parental education and the age at first sexual intercourse.

## 4. Discussion

In this study, sexual risky behaviors in T1D adolescents in comparison with their healthy peers are examined. To the best of our knowledge there are very limited previous studies regarding sexual activity as well as risky behaviors in T1D adolescents, and most of them did not include control populations [20,21].

### 4.1. Sexual Behavior

Chronic disease may affect children’s transition through adolescence in many ways, including anticipated sexual exploration. In this study, a significantly lower percentage of T1D adolescents reported sexual experience than their healthy peers (74.1% vs. 87.4%, *p* = 0.033). In contrast, reported sexual intercourse was similar in both groups (49.5% for the adolescents without T1D, and 45.1% for the T1D group).

Consistent with our findings, previous U.S. studies [20,21,26] and Giraudo et al. [27] have shown a lower prevalence of sexual activity among T1D adolescents compared to peers without T1D, suggesting increased cautiousness and hesitance regarding sex. Of note, the overall prevalence of sexual activity in our study was higher than that reported in the above studies (18–24%), probably due to population differences.

Alcohol use is the most frequently reported risky behavior among T1D adolescents with 30% reporting a drinking episode in the past year [21]. However, the rates of alcohol use observed in this population were below the range of 42–71.8% previously reported for 10–16 year old peers without T1D [21,28,29]. In addition, in the Jacobson et al. study [30], fewer T1D adolescents used alcohol, tobacco or marijuana than the comparison group. Although moderately heavy alcohol consumption by adults with T1D has been associated with hypoglycemic episodes and hypoglycemic unawareness [12], in T1D adolescents it has only been associated with glycemic variation [31].

In 2009, the Centers for Disease Control (CDC) and Prevention’s Youth Risk Behavior Surveillance reported that 72.5% of U.S. high school students had consumed alcohol, while 21.6% had drunk alcohol or used drugs prior to the last sexual intercourse [7]. Of note, the use of alcohol before sex has negative effects on the adolescents’ judgment and inhibitory control [32]. In the present study, alcohol consumption before sexual intercourse was similar for both groups (T1D 13.0% vs. comparison group: 19.6%), and these proportions were similar to those reported by the CDC; however, intoxication by alcohol prior to sexual intercourse was reported four times less frequently in T1D adolescents than adolescents without T1D (4.3% vs. 20%, *p* = 0.01), and all cases were boys. As previously shown in the general population [23], girls used alcohol more judiciously than boys. To the contrary, although a study of adolescents in Italy [33] showed less frequent alcohol consumption among females, males with T1D consumed alcohol less frequently than females.

In this study, overall risky behaviors were observed less frequently among adolescents with T1D than adolescents without T1D (8.62% vs. 23.27% respectively). This has also been previously shown in other populations [21]. In Martinez-Aguayo et al. [34], risky behaviors were observed less frequently in younger adolescents with T1D but in older adolescents with T1D, the prevalence of risky behavior was the same. This may be attributed to diabetes management education, delayed personal social development and initiation of risk-taking behaviors. To the contrary, similar sexual risky behaviors were found in a study of adolescents and young women with T1D [27].

In our study, adolescents with T1D and ≥2 risky behaviors were all boys, at an older age and younger at first sexual intercourse than their peers without T1D. The frequency of risky behaviors among adolescents with T1D has been previously shown to increase with age [21,34]. These findings suggest that adolescents with T1D and especially boys, engage in risky behaviors similarly to their healthy peers, despite the negative health effects on diabetes management, perhaps because they do not wish to differ from their peers.

In accordance with our findings, a lower frequency of risky behaviors among girls with T1D has been previously described and attributed to the fear of unplanned pregnancy and its consequences in relation to the presence of diabetes [21]. However, regarding our cohort of adolescents with T1D, parental overprotection and strict moral issues characterizing Greek parents, may significantly prevent adolescents with T1D and especially girls from risky behaviors.

Τhe majority of adolescents without T1D with ≥2 risky behaviors in our study were males and of older age. No significant difference was observed in terms of parental education and the age at first sexual intercourse between the two groups of adolescents without T1D. Similarly, in a previous study from Greece on healthy youths, proportionally more boys were sexually active and had their first sexual experience or intercourse at an earlier age compared to girls. However, girls had their first sexual intercourse with an older partner and reported no use of contraception more frequently than boys [23]. Similarly, girls in both groups of our study were more cautious about risky behaviors than boys were, and adolescents with T1D were more cautious compared to their peers without T1D, possibly due to the consequences of risky behaviors (alcohol and tobacco consumption, unprotected sex) and oral contraceptive use on diabetes management and complications [35,36,37]. Furthermore, in agreement with our study, in the study by Giraudo et al. [27] on 115 adolescents and young women with T1D and 386 peers without T1D it was found that fewer adolescents with T1D than controls had sexual activity. Thus, adolescents and young women with T1D were more cautious than their peers in terms of risky sexual behaviors. However, in the latter study a significant proportion of adolescents and young women with T1D smoked or used illicit substances, which did not occur in our study, and a significantly higher proportion of participants had unprotected sex in comparison with those of our study. These differences could be attributed to the older age range of the study population in the Giraudo et al. study [27].

In the present study, a small number of adolescents with T1D with a risky behavior had a higher maternal education and were younger at sexual debut than those in the comparison group. Although low income and minority youth are often considered at greater risk for drug and alcohol use, studies indicate that white adolescents living in suburban or middle-class households are actually more likely to drink alcohol and use illicit drugs [38]. Regarding sexually risky behaviors in adolescents without T1D, previous studies have not identified any association with household socioeconomic status [13,39,40]. In only one study, timing of sexual debut among 16-year-old boys in Oslo [41] presented a U-shaped pattern of parental social class, with upper managerial and manual working classes having similarly high proportions of early debutants. These findings are in agreement with our study, but they need to be interpreted cautiously, especially due to the small number of early debutants with T1D in our study; thus, larger relative studies are needed to elucidate these associations.

No significant difference in diabetes duration and glycemic control between adolescents with or without risky behaviors was observed in our study. It has been previously reported that adolescents with chronic conditions may be even more likely to engage in high risk behaviors than their peers [42], including early sexual activity, and these behaviors have an adverse effect on glycemic control [13,33,42]. A significantly higher rate of poor glycemic control among adolescents engaged in risky behaviors has been previously reported [33]. Moreover, in the Giraudo et al. study [27], poor glycemic control was associated with high risk sexual behaviors.

In the present study, adolescents with T1D with ≥2 risky behaviors were mostly boys with younger age at sexual debut. Thus, it has been suggested that counseling on sexual matters in adolescents with T1D should begin early in puberty, in order to give proper preconceptional education and possibly delay sexual debut [27].

### 4.2. Strengths and Limitations

A main strength of this study was the inclusion of a 2:1 matched control group. Moreover, comparison with sexual behaviors of a sizable Greek adolescent population was possible [23].

Among the limitations of our study was the presence of a relatively small number of adolescents with risky behaviors and the possible bias resulted by the use of self-reported data. Another limitation of the present study was that the study sample was relatively small; however, it was uniform (as all patients had a Greek origin) and it was representative of the Greek adolescent population, as it comes from a tertiary Children’s Hospital, which is a reference center for central and southern Greece and the islands.

## 5. Conclusions

The analysis of the present data shows fewer adolescents with T1D had a sexual experience in comparison with their peers. Moreover, adolescents with T1D were engaged in risky sexual behaviors less frequently than controls and girls less frequently than boys. Characteristics of adolescents with or without T1D with ≥2 risky behaviors were older age and predominantly male gender, while characteristics of solely adolescents with T1D and ≥2 risky behaviors were younger age at first intercourse and higher maternal education, with no difference in HbA1c or diabetes duration. In conclusion, adolescents with T1D and especially girls were more cautious in terms of risky behaviors than their peers were. However, a small proportion of adolescents with T1D, especially boys, might engage in high risk sexual behaviors, such as unprotected sex, sex with multiple sexual partners, or alcohol consumption before sex, which bear potential significant adverse health effects, such as undesired pregnancy, sexually transmitted diseases or unexpected hypoglycemia.

Based on the above findings, health care providers and diabetes educators should assess for risk behaviors in adolescents with T1D, bearing in mind the gender difference in risky sexual behavior, and provide patient and parental counseling at early puberty to prevent relevant adverse health effects. Furthermore, the population of adolescents with T1D merits future studies to develop and implement evidence based primary prevention programs.

## Figures and Tables

**Table 1:** Sexual activity of the two study groups.

	Group		
	Adolescents without T1D	Adolescents with T1D	*p*
Sexual experience, (%)	97 (87.4)	40 (74.1)	0.033 *
Age at first sexual experience, mean (SD)	14.7 (1.5)	14.5 (2.3)	0.647 ^+^
Sexual intercourse, (%)	54 (49.5)	23 (45.1)	0.600 *
Age at first sexual intercourse, mean (SD)	15.2 (1.5)	15.9 (1.8)	0.133 ^+^

Abbreviations: T1D = Type 1 diabetes mellitus, SD = standard deviation score. * Pearson’s chi square test; ^+^ Student’s *t*-test.

**Table 2:** Risky behaviors in sexually active adolescents for the two groups.

	Groups		
	Adolescents without T1D	Adolescents with TID	
	N (%)	N (%)	*p*
First sexual intercourse with older partner	34 (29.3)	18 (31.0)	0.265 *
Age difference between sexual partners			
Less than one year	15 (34.9)	9 (47.4)	0.519 **
One to five years	19 (44.2)	9 (47.4)	
Six to ten years	8 (18.6)	1 (5.3)	
More than 10 years	1 (2.3)	0 (0.0)	
Alcohol consumption before first sexual intercourse	10 (19.6)	3 (13.0)	0.743 **
Alcohol consumption by the sexual partner before the adolescent’s first sexual intercourse	10 (19.2)	3 (13.6)	0.743 **
Alcohol consumption before any sexual intercourse	18 (35.3)	7 (30.4)	0.683 *
Alcohol consumption by the sexual partner before any sexual intercourse	15 (30.0)	8 (34.8)	0.683 *
Having ever been drunk before a sexual intercourse	10 (20.0)	1 (4.3)	0.0156 **
The partner being drunk before a sexual intercourse	7 (14.6)	2 (9.1)	0.709 **
No of sexual partners			
1	20 (42.6)	7 (31.8)	0.171 **
2	7 (14.9)	8 (36.4)	
3	4 (8.5)	0 (0.0)	
4	5 (10.6)	4 (18.2)	
>4	11 (23.4)	3 (13.6)	
Forced to have sex against their will	8 (15.7)	1 (4.3)	0.258 **
Unprotected sex	7/37 (18.9%)	1/15 (6.66%)	0.339

Abbreviations: T1D = Type 1 diabetes mellitus. * Pearson’s chi-square test; ** Fisher’s exact test.

**Table 3:** Analysis of risky behaviors by gender.

	Groups		
	Boys	Girls	
	N (%)	N (%)	*p*
(A) ADOLESCENTS WITHOUT T1D			
First sexual intercourse with older partner (≥6 years older)	8 (88.8)	1 (11.1)	0.139 *
Alcohol consumption before the first sexual intercourse	10 (100)	0 (0)	0.025 *
Forced to have sex against their will	5 (62.5)	3 (37.5)	0.591 *
Unprotected sex	5 (71.4)	3 (37.5)	0.591 *
Sex with multiple (≥4) partners	10 (90.9)	1 (9.0)	0.128 *
(B) T1D ADOLESCENTS			
First sexual intercourse with older partner (≥6 years older)	1 (100)	0 (0)	0.621 *
Alcohol consumption before the first sexual intercourse	6 (100)	0 (0)	0.739 *
Forced to have sex against their will	1 (100)	0 (0)	0.739 *
Unprotected sex	1 (100)	0(0)	0.867 *
Sex with multiple (≥4) partners	3 (100%)	0 (0)	0.426 *

Abbreviations: T1D = Type 1 diabetes mellitus. * Fisher’s exact test.

**Table 4:** Characteristics of T1D adolescents according to the presence/absence of two or more risky behaviors.

		Risky Behaviors				
		Absent (Ν = 36) (60.34%)		Present (Ν = 5) (8.62%)		
		Ν	%	Ν	%	*p*
Age, mean (SD)		16.2	2.2	17,8	1.0	<0.031 **
Gender	Males	18	78.3	5	21.7	0.045 ***
	Females	17	100	0	0	
HbA1c, mean (SD)		8.1	1.3	7.9	1.0	0.743 **
Duration of disease, mean (SD)		6.8	3.1	6.9	2.6	0.931 **
Age at the time of diagnosis, mean (SD)		9.3	2.7	9.9	2.2	0.662 **
Father’s educational status	Primary/Middle school	7	21.2	1	20	0.906 *
	High/Technical school	16	48.5	2	40	
	University/Postgraduate studies	10	30.3	2	40	
Mother’s educational status	Primary/Middle school	9	27.3	0	0	0.039 *
	High/Technical school	14	42.4	1	20	
	University/Postgraduate studies	10	30.3	4	80	
Age at first sexual experience, mean (SD)		14.6	2.4	13.8	1.8	0.481
Age at first sexual intercourse, mean (SD)		16.3	1.9	14.8	0.8	0.031 **

Abbreviations: T1D = Type 1 diabetes mellitus, SD = standard deviation scores, HbA1c = glycosylated hemoglobin A1c. * Pearson’s x^2^ test, ** Student’s *t*-test, *** Fishers’ exact test.

**Table 5:** Characteristics of adolescents without T1D according to the presence/absence of two or more risky behaviors.

		Risky Behaviors				
		Absent (Ν = 67) (57.7%)		Present (Ν = 27) (23.2%)		
		Ν	%	Ν	%	*p*
Age, mean (SD)		15.7	1.2	16.5	1.4	<0.006 **
Gender	Males	28	56	22	44	0.001 ***
	Females	40	88.8	5	11.1	
Father’s educational status	Primary/Middle school	11	19.3	5	19.2	0.532 *
	High/Technical school	32	56.1	12	46.1	
	University/Postgraduate studies	14	24.5	9	34.6	
Mother’s educational status	Primary/Middle school	13	22.8	6	23	0.601 *
	High/Technical school	27	47.3	7	26.9	
	University/Postgraduate studies	22	38.6	12	46.1	
Age at 1st sexual experience, mean (SD)		14.8	1.8	14.4	1.3	0.220
Age at first sexual intercourse		15.6	1.1	14.8	1.7	0.120 **

Abbreviations: T1D = Type 1 diabetes mellitus, SD = standard deviation score, No=patients’ number. * Pearson’s x^2^ test, ** Student’s *t*-test, *** Fishers’ exact test.

## Data Availability

Data is contained within the article.
